# Neural dynamics and network topology interact to form critical avalanches

**DOI:** 10.1186/1471-2202-12-S1-P118

**Published:** 2011-07-18

**Authors:** Anna Levina, J Michael Herrmann, Theo Geisel

**Affiliations:** 1 Max Planck Institute for Mathematics in the Sciences, Inselstr. 22, Leipzig, D-04103, Germany; 2 School of Informatics, IPAB & ILSI, University of Edinburgh, 10 Crichton St, EH8 9AB, Scotland, UK; 3 MPI for Dynamics and Self-Organization, Bunsenstr. 10, Göttingen, D-37073, Germany; 4 BCCN Göttingen, Bunsenstr. 10, Göttingen, D-37073, Germany

## 

Self-organized criticality (SOC) is one of the key concepts for describing the emergence of complexity in nature. In neural systems, the critical state is believed to optimize memory capacity, sensitivity to stimuli and information transmission. Critical avalanches were found in cortical cultures and slices [1] and in the motor cortex of awake monkeys [2]. Computational models of SOC often include an explicit regulatory mechanism that guides the state of the network toward criticality. We have shown previously [3,4] that synaptic facilitation and depression are sufficient to explain the self-organization of critical behavior in a network of non-leaky neurons. This model lead to the prediction of an activity-dependent switching mechanism for up and down state dynamics in prefrontal cortex [4].

Models of neural avalanches may include mechanisms on neural, synaptic or network level. In the present contribution we propose a generalized model that combines short-term synaptic dynamics with homeostatic effects that are controlled on the neural level as well as long-term plasticity that causes change in the network structure. We show that the interaction of these effects is indeed constructive in the sense that the critical state of the network is stably maintained.

We also studied how criticality influences learning in neural networks and vice versa how the network can maintain criticality in face of a changing topology. While e.g. strong random dilution of the connectivity may initially induce a subcritical behavior, criticality is quickly reinstalled by a local learning rule that, in this case, affects essentially only the synaptic rescaling. The learning rule is homeostatic: it aims to stabilize the postsynaptic response to the spiking activity of the neuron. More complex network structures are either fully compatible with SOC (such as small-world topologies that were found in critical network reconstruction [5]) or require a substantial reorganization of the network. The latter is observed e.g. in networks with nearest neighbor connections that do not attain criticality for any synaptic strength, but are transformed into a critical network by growth of a small number of additional connections.

The adaptation rules that bring about SOC are also compatible with other types of learning e.g. for the formation of memories. Here again, homeostatic learning compensates the structural effects of learning in the system. This also allows us to study how STDP shapes the critical network by self-organization. We have thus provided a framework that represents a mechanism of SOC in a general sense and that can be used for testing the impact of various neurotransmitters, for the integration of senso-motoric loops and consolidation of memories. The essential interaction in multi-level learning extends past results that were based on the functional independence of neural and network dynamics.
